# Qualitative Exploration of Barriers to Medication Adherence Among Patients with Uncontrolled Diabetes in Saudi Arabia

**DOI:** 10.3390/pharmacy9010016

**Published:** 2021-01-11

**Authors:** Ghaida Alodhaib, Imtinan Alhusaynan, Ahmer Mirza, Yasser Almogbel

**Affiliations:** 1 Department of Pharmacy Practice, College of Pharmacy, Qassim University, Buraidah, Qassim 51452, Saudi Arabia; ghaidaalodhaib@hotmail.com (G.A.); emtenanf.94@gmail.com (I.A.); ah.mirza@qu.edu.sa (A.M.); 2 Department of Applied Sciences, University of Huddersfield, Queensgate, Huddersfield HD1 3DH, UK

**Keywords:** diabetes mellitus, uncontrolled, medication adherence, barriers, Kingdom of Saudi Arabia, qualitative

## Abstract

Uncontrolled diabetes is associated with macrovascular and microvascular complications that compromise the quality of life; however, the patients’ perspectives about medication non-adherence are unclear. We aimed to understand patient behavior and explore the barriers to medication adherence in uncontrolled diabetes patients. We employed a qualitative method of face-to-face interviews conducted with adult patients in Saudi Arabia who had uncontrolled diabetes mellitus (glycosylated hemoglobin >7% or fasting blood glucose >7.2 mmol/L). All interviews were audio-recorded and analyzed using thematic analysis. The interviews were conducted for 68 patients. Sixty-seven patients were suffering from Diabetes Mellitus Type 2, and one patient was suffering from Diabetes Mellitus Type 1. We identified the barriers to medication adherence and classified them under six main factors: patients-, medications-, healthcare-, provider-, social-, and disease-related factors. The main barriers identified were the use of alternatives, hard-pressed for time, polypharmacy, bad relationship with the physician, cultured beliefs, self-alteration of the dose, exposed side effects, ineffective medications, refusal of insulin, multiple doctor visits, uncontrolled diet, and forgetfulness. Multiple barriers that prevented the patients from medication adherence were related to poor knowledge, counseling, psychological management, and social support. Appropriate educational programs, suitable patient-specific counseling, and close follow-ups would be required to improve the knowledge, outcomes, and quality of life in uncontrolled diabetes patients.

## 1. Introduction

Diabetes mellitus (DM) is a chronic metabolic disease caused by the lack of insulin and impaired insulin secretion [1]. The prevalence of diabetes has increased rapidly over the years [2]. In 2019, an estimated 9.3% of the world population, or 463 million people in all age groups, had DM. This number is expected to increase to 10.2% or 578 million people in 2030 [3]. The abnormal values that are indicators of diabetes are fasting blood glucose (FBG) ≥ 126 mg/dL (7.0 mmol/L), 2-h plasma glucose (PG) ≥ 200 mg/dL (11.1 mmol/L) during oral glucose tolerance test (OGTT), glycated hemoglobin (HbA_1_c) ≥ 6.5%, and classic symptoms of hyperglycemia with random blood glucose ≥ 200 mg/dL (11.1 mmol/L) [4].

Uncontrolled diabetes can lead to several complications of DM, categorized as macrovascular and microvascular complications. Macrovascular complications affect large blood vessels, the most common ones being coronary artery disease and stroke. Microvascular complications affect small blood vessels, such as those supplying blood to the eyes and kidneys, and can cause nephropathy, retinopathy, and neuropathy. The prevalence of diabetic nephropathy, retinopathy, and neuropathy in Saudi Arabia is 33.3%, 19.7 %, and 19.9%, respectively [5,6,7]. Uncontrolled diabetes can lead to these complications if people fail to manage their blood glucose levels, which hurts life quality. Globally, medication non-adherence is a major contributor to treatment failure because it influences medication action and effectiveness [8,9].

Nonadherence is defined as the patients’ noncompliance with healthcare providers’ instructions or recommendations regarding their treatment plans, including medication use or lifestyle changes, ultimately affecting the outcomes [9,10]. According to the World Health Organization, the rate of adherence to chronic disease therapies in developed countries is about 50%, and this rate is even lower in developing countries. The low adherence rate represents our need to identify medication adherence barriers in diabetes patients [11].

Previous studies focused on uncontrolled diabetes patients’ perspectives and their healthcare providers in identifying the barriers to medication adherence. For example, the healthcare providers observed that aging patients would forget to take their medications, informed patients could understand the purpose of adherence, some patients could not buy the medications, and incorrectly took their medications with insufficient time for the healthcare providers to explain the correct usage. In contrast, patients disclosed that they changed their medication dose when fasting, would not use the medications, did not believe that the medications affected maintaining blood glucose, and would be healthy without medications when returning to their home country [12]. Many of the barriers identified were directly related to patient information and knowledge about diabetes and its management. These would require concerted efforts and a series of interventions, which should be initiated in a step-wise approach. Future work should focus on creating population-specific interventions, taking into consideration the barriers identified in this study. Without overcoming medication adherence barriers, it is improbable to improve patient outcomes among patients with uncontrolled diabetes. [12].

We need to understand the patients’ perspectives to provide them with high-quality and effective services and valuable healthcare interventions. Physicians can rarely identify non-adherent patients, their problems with medications, and their beliefs regarding medications; instead, they rely on laboratory tests to determine the therapies’ success. However, these results are not indicative of medication adherence. Using qualitative methods such as interviews and surveys of the patients about their medication behaviors, we can understand their beliefs and behaviors and recognize their reasons or barriers for non-adherence to their antidiabetic medications [11].

In this study, we aimed to explore the barriers encountered by the patients with uncontrolled diabetes leading to poor adherence to medication by integrating the patients’ perspectives in Saudi Arabia.

## 2. Materials and Methods

We conducted a qualitative study using face-to-face interviews with patients diagnosed with DM (type 1 and type 2) at a diabetes center, frequently used this study in health services research to describe patients’ experience. The Ministry of Health in Saudi Arabia approved this study (1440-1411949). 

### 2.1. Patient Selection

We conducted this study with 71 adults (age ≥ 18 years) diagnosed with DM for at least one year. They had HbA1c > 7% or FBG > 7.2 mmol/L and had visited a doctor at the diabetes center. The test result was collected 24 h before the interview. We reviewed their medical records to identify the inclusion criteria. We excluded three patients who did not speak either Arabic or English. All patients interviewed were registered at the Abdullah Suliman Al-Bassam for Diabetic Center in King Saud Hospital (KSH) in Qassim, Unaiyzah, Saudi Arabia. Patients were recruited using a convenience sampling method.

### 2.2. Study Design

We prepared an interview guide based on grounded theory for the collection of the following data: (1) demographic data, (2) laboratory findings (HbA_1_c, RBG, and FBG), (3) patient-related factors, (4) medication-related factors, (5) social-related factors, (6) provider-related factors, (7) healthcare-related factors and (8) disease-related factors.

### 2.3. Data Collection 

The face-to-face interviews with the patients were conducted in either English or Arabic based on their preferred language. First, we introduced the participants to the interviewer and explained the purpose of the interview. Second, we obtained informed consent from them to assure them of the privacy of the interview. Third, based on the question model, we asked open-ended questions, such as the difficulties they encountered that affect their blood glucose regulation. The interview question model covered six categories of factors contributing to poor adherence to the medications: patients-, medication-, disease-, provider-, healthcare-, and social-related factors. Patients who did not provide complete answers about these factors in the open-ended questions were asked close-ended questions. The interviews were recorded to ensure accuracy. We continued the interviews with the participants until every aspect of their barriers were covered. We deemed 60 participants with uncontrolled diabetes sufficient to reach saturation for this study. Secondly, it depends upon the number of patients visiting the Diabetic clinic in that hospital during the mentioned period and meets the criteria for inclusion in the study. The study was conducted between 13 March and 3 April 2019, until all 60 patients were interviewed.

### 2.4. Data Analysis

The collected data were analyzed manually through thematic analysis, a technique commonly used in qualitative studies. All interviews with the patients were audio-recorded, and every patient was provided with a unique code manually. We then translated these recordings from Arabic to English. All the questions and answers of every patient were tabulated with their patient codes. We interviewed the patients until data saturation was attained. Then, we identified the similar barriers encountered by many patients, along with some unique answers. Next, we consolidated the results with quotations from some patients.

## 3. Results

During the study period, 1071 patients with diabetes visited the center, of which 25.4% (273) were males, and 74.5% (798) were females. Of these, 185 patients had uncontrolled diabetes, with 34.5% (64) males and 65.4% (121) females. We conducted interviews with 71 patients comprising 26 males and 45 females. Three patients were excluded: one was afraid to speak anything, another spoke rashly, and the last one was controlled. The mean duration of the interviews was 22.5 ± 5 min. The mean age of the patients with uncontrolled diabetes was 52 years, and the mean HbA_1_c and FBG levels were 9.44% and 12.35 mmol/L, respectively. The mean duration of diabetes was 12 years (1.47%) of them have type 1 diabetes. Whereas (98.53%) have type 2 diabetes. The average age was 54.8 ± 12.2 (Table 1). The majority were Saudis (98.5%) females (64.7%) participants. Most participants reported their education level as illiterate (32.4%), followed by primary school (19.12%) and high school (19.12%). Besides, most of them had a medium-income (64.7%). Of the 68 patients, 36 had neuropathy, 32 had retinopathy, and none had nephropathy. The patients’ various diabetic medications included metformin, linagliptin, sitagliptin, gliclazide, insulin glargine, insulin aspart, insulin lispro, and regular insulin.

### 3.1. Barriers to Medication Adherence

**Figure 1:**
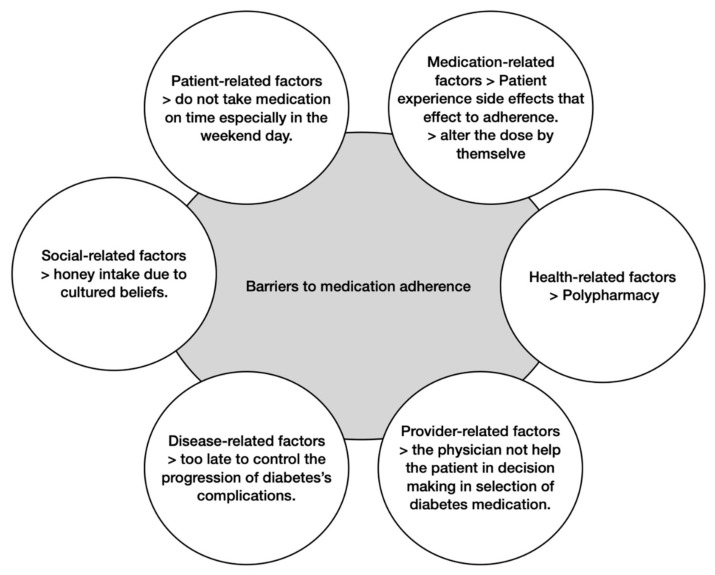
Common barriers to medication adherence.

Six main categories were elicited from the interviews with patients. Figure 1 represents some of the factors and main barriers that we identified.

#### 3.1.1. Patient-Related Factors

Patients’ characteristics: We recognized several individual characteristics of the patients, including age, education level, and financial state, as significant barriers to medication adherence.

Few patients had low financial status and could not afford blood glucose monitoring devices to measure their blood glucose levels at home.

[…] my blood glucose device is old, and when I ordered it from the hospital, it was not available, and I could not buy it. So, I did not know my blood glucose levels during the day. [Pt No. 50].

When asked if their medications are convenient to them, the older individuals tended to have lower adherence because they were bored to take the medications than that in younger adults.

[…] I am bored because I am taking it for a long time. [Pt No. 49].

Patients’ perceptions, attitudes, and behaviors: The patients’ attitude with wrong perceptions of the disease affected their medication adherence. Many of them had inadequate knowledge regarding medications for their health and the consequences of non-adherence. 

Some patients had difficulty believing that diabetes is an autoimmune or genetic disease. They instead believed that it is a psychological condition.

[…] I believe the cause of my disease is psychological, and due to stress related to work. When I will quit my work, I will cure. [Pt No. 58].

Some patients believed that exercise and diet were adequate to control blood glucose levels, and there was no need to take the medications, even if the blood glucose level remained higher than normal.

[…] Diabetes started 27 years ago, and I started taking medication after 20 years because I did not believe in the drugs. I replaced them with diet and exercise, but I was wrong. [Pt No. 58].

Some patients did not want to be dependent on drugs. Therefore, they used to take medications while searching for alternatives.

[…] I tried cinnamon in water, and I saw its benefit, thus reducing the dose. [Pt No. 36].

[…] I try to use olive oil, but I cannot leave the drugs yet. [Pt No. 42].

Another patient ate a lot of sugar, especially during weekends, while forgetting to take the medications. 

[…] on the weekend, there is a family gathering, I ate a lot of sugar with them, and I forgot to take the medications. [Pt No. 15].

Many patients skipped the medicine intentionally when they measured their blood glucose levels at home and found them to be low.

[…] I did not take it intentionally and use metformin like a regulator. When my blood glucose increases, I take it, and if not, then I leave it. [Pt No. 59]. 

Many patients tried the medications for a long time and were not satisfied because they did not see any effects. They could not believe that the medications regulate their blood glucose or improve the outcomes. They believed that the drug’s main purpose is to make pharmaceutical companies’ profits and not benefit patients. 

[…] I have not seen any benefit from metformin. Hence, I do not take it every day because I am not satisfied with my medication. I cannot believe it regulates my blood glucose. [Pt No. 7]. 

[…] metformin is useless and ineffective. It just enters my body and exits without any benefits. As a clinical pharmacist, I think that you should do more tests on the effects of metformin. [Pt No. 58].

Few patients neglected themselves as their family members were extremely sick, affecting their own medication adherence.

[…] In the past, I have traveled a lot because my husband and daughter had paralysis, and I felt responsible and went with them for hospital appointments in another country. Sometimes my medicine was over, so I visited another hospital, and they would dispense it to me. [Pt No. 50].

#### 3.1.2. Medication-Related Factors

The medications were an important factor and highly affected the adherence of the patients. The patients tended to adhere to their medications if it was convenient for them, and vice versa.

[…] I am not satisfied with my medications because they cause skin swelling. [Pt No. 47].

They reduced the dose or stopped taking oral medications by themselves because they could not tolerate it or experienced nausea, vomiting, cramping, stomach pain, and hypoglycemia.

[…] I cannot tolerate metformin. I experience nausea, cramping, and vomiting. Based on the severity of pain, either I reduce the dose to one tablet instead of three or not take it. [Pt No. 3]. 

[…] I do not follow the instructions in taking the medications. The doctor prescribed 40 units to be taken before breakfast, which caused hypoglycemia, so I changed it to 35 units. [Pt No. 62].

#### 3.1.3. Social-Related Factors

Several patients kept trying different traditional alternatives that other people suggested to them or heard from doctors on social media outlets with or without medication, wishing to cure DM and stop the medication over time. 

[…] Someone told me about how they drank “Barley coffee” for one year, and then the doctor said that they were cured of diabetes and stopped their medications. [Pt No. 1].

[…] Doctors on social media said that *Nigella sativa* is very helpful in reducing blood glucose. I tried it and saw the benefit, but I stopped exercising. That may be the reason for the current increase in my blood glucose. [Pt No. 32].

Some patients reduced or stopped their medication because of fear of kidney or liver failure after hearing the doctors on social media. 

[…] I usually stop the medication every other week because I am afraid it may hurt my kidneys. [Pt No. 4].

[…] Many people told me that these medications lead to liver failure. [Pt No. 10].

Some patients could not take insulin by themselves and had no one to take care of them.

[…] I cannot take the insulin by myself; my daughter administers it to me. At night, she goes home, and there is no one to administer insulin. [Pt No. 21].

Few patients felt neglected by the people in their surroundings, as they were not supportive and motivating. This affected their medication adherence.

[…] My friends have misconceptions about diabetes and differentiate me because I have it. They joke in a bad manner with me, which affects my mood. Subsequently, I stop caring about my insulin, which is why my blood glucose is not controlled. [Pt No. 36].

#### 3.1.4. Healthcare-Related Factors

The older patients were mainly covered in these factors because they suffered from multiple diseases and took many drugs. This results in polypharmacy, where the patient taking many drugs has increased chances of side effects and forgets to take diabetes medications. 

[…] Usually, I forget to take diabetes medications because I have many other medications and diseases. [Pt No. 64]. 

Some stopped taking medication or took an old medicine because their medication was finished and no one could bring it for them, or they could not find it easily. 

[…] If my medications were over, and no one could bring it to me, or I could not buy it from any nearby pharmacy, I would take previous antidiabetic medication that was stored at home. [Pt No. 4]. 

#### 3.1.5. Provider-Related Factors

Many patients went to more than one physician for diabetes, considering different advices and medications, and creating confusion for themselves.

[…] I do not have a relationship with the physician because I visit a different one every time. [Pt No. 39 and 40].

The doctor did not support the patient during improvement, making the patient depressed, and not caring about medication. 

[…] The doctor did not support me when I told him that I registered in the Gymnasium. He said that even if I underwent a sleeve gastrectomy, I would not cure my diabetes. Every time I remember that day, I feel depressed and forget medications. [Pt No. 37].

One patient suffered from diabetes medications and experienced many complications, as the consultant did not prescribe appropriate medication. The patient lost their trust in the physician, affecting their medication adherence.

[…] I have complications, such as numbness, feeling hot in the legs and arms, and fungal infection on my foot; all of these are because of my physician not consulting good drugs, causing my disease to progress rapidly. […] I feel that the physician is not good, and I do not trust their knowledge anymore. [Pt No. 65].

#### 3.1.6. Disease-Related Factors

Many patients suffered from different complications due to diabetes (Table 1). They stopped taking the medications or decreased their adherence because they assumed it was too late to control the disease’s progression.

## 4. Discussion

This is the first study conducted in Saudi Arabia to identify the barriers to medication adherence among patients with uncontrolled diabetes based on their perspective, using a qualitative methodology. Previous studies were conducted in Qatar and North Africa [12,13]. To improve their medication adherence, we need to understand the patients’ reasons and barriers that lead them to become non-adherent. 

In this study, we identified many barriers, such as individual characteristics of the patients, older age, and financial status, consistent with the findings of Jaam et al. [12]. In their study, Jaam et al. identified financial status as one of the barriers because some patients had difficulty paying for their medications. In Saudi Arabia, healthcare is currently available free of charge to all Saudi citizens and expatriates working in the public sector, mainly through the Ministry of Health and supported by other public health facilities [14]. All government hospitals provide medications for free. Patients in all the financial brackets, even those who were affluent, preferred to obtain their medications from government hospitals; thus, the financial status in Saudi Arabia may not be a significant factor. This study also corroborates other studies indicating that patients find it difficult to pay for the glucometer because of the hospital’s limited capacity. Besides, Jaam et al. found that older patients exhibited lesser adherence than younger patients. Our study confirms the same; this is possible because older patients have suffered multiple diseases for a long time and are not inclined to take medications [12].

From studying the patients’ perceptions, attitudes, and behaviors in our study, we observed that one of the barriers was their inaccurate belief that the causes of the DM were psychological. Because of this belief, the patients did not take their medications; they assumed that quitting their work would relieve the stress, and the disease would disappear. Additionally, we found other psychological conditions, such as depression, overthinking, and self-neglect, that affected medication adherence. Healthcare providers also play a major role in the patient’s psychology by motivating them and discussing the best available medication. In our study, some patients did not care about the medication because they felt neglected and did not receive appropriate care from their healthcare providers [15,16].

Another barrier was that the patients believed that they could replace DM medications or not use them because they did not want to get dependent on drugs. They assumed that diet and exercise were adequate to control blood glucose levels or preferred to use traditional alternatives instead of taking the prescribed medications due to their fear of side effects on their bodies; these factors were also highlighted by Jaam et al. [12]. This approach is not effective for most patients because DM cannot be controlled without medication.

We also found that patients would adjust the dose of insulin without consulting their physicians, especially when they were worried regarding the side effects or kidney and liver problems due to medications, which is again in agreement with the findings of Jaam et al. [12]. Some patients spent their time with their families on weekends, often forgetting to take their medications. However, most people relied on alternative therapies to reduce their blood glucose levels. Some people intended to reduce or stop their medications after the benefits of such alternative therapies appeared, indicating their lack of education and knowledge about chronic disease and its management [17].

One of the barriers was the perception of some patients regarding the effectiveness of the drugs. They believed that the medications were not effective and only increased pharmaceutical companies’ profits, a point also highlighted by Jaam et al. [12].

The common barriers to medication adherence are polypharmacy because DM patients, especially the older ones, suffer from multiple diseases and take many medications for these diseases. Such patients tend to forget their medications. 

Several methods and strategies exist to improve medication adherence in patients with DM [15,16,17,18,19,20]. Additionally, there are published studies on physicians’ roles in improving medication adherence, such as medication therapy management, telemonitoring program, education, and follow-up [21,22]. Patient education is important to emphasize the need for adherence to medications and the instructions and time of taking medication on normal days, in the Ramadan month, and during traveling. Additionally, the physicians should rectify patients’ misconceptions regarding the side effects of the medications and any complications. They should encourage and educate those who refuse to take insulin about its benefits and teach the patients to adjust the dose of insulin or to use their cellular phones to adjust insulin doses, as exhibited in the study of Alotaibi et al. The physicians should also encourage the patients to test their blood glucose levels daily [17,23].

## 5. Strengths and Limitations

This study provides a real insight into the current status of medication adherence rates among patient with type 2 diabetes mellitus (T2DM) in Saudi Arabia. Adherence can be measured by different methodological approaches. In this study, adherence was assessed using the Qualitative direct patient interviewing method, which is a reliable method and depends on real-world data obtained from patient. This method can be used for a large population at a relatively low cost and also prevents recall bias, which is associated with patient interview-based adherence methods. This study sheds some light into concomitant diseases that were associated with the non-adherence. Although the current study provides real-world information from more than 68 patients about adherence rate to different anti-diabetics drugs and the relationship with glycemic control, few limitations were observed. The study population was mostly patients who attended the Diabetic Center in central Saudi Arabia; therefore, it is difficult to extrapolate the study findings to the whole Saudi population residing in the other geographical areas of the country. Nevertheless, patients included in this study represent mostly Saudi citizens and a few expatriate groups living in Saudi Arabia. A convenience sampling method was used, the possible influence of non-respondents on our study could not be identified. Findings from this study cannot be generalized to different populations and settings. 

## 6. Conclusions

This study exhibited the barriers to medication adherence encountered by patients with uncontrolled DM in the Qassim region of Saudi Arabia. Many diabetic patients do not achieve glycemic control because of poor adherence, which leads to serious complications, such as cardiovascular diseases. Those complications would impact the healthcare at the patient level and institutional level, putting more load over the shoulder regarding the cost. Uncontrolled DM needs more attention and concerted efforts to explore all the possible barriers and improve patient adherence. Considering the barriers identified in this study, we can conclude that non-adherence is associated with poor knowledge, lack of counseling, shortage psychological management, and social support. Interventional studies are crucial to appropriately evaluate controlling adherence berries to improve the overall outcomes.

## Figures and Tables

**Table 1:** Characteristics of Patients with Uncontrolled Diabetes Mellitus, Who Participated in Qualitative Interviews (N = 68).

Characteristic	Number of Patients (*n* = 68)
Age (years); mean ± SD	54.8 (±12.2)
Gender	
Male, *n* (%)	24 (35.29)
Female, *n* (%)	44 (64.71)
Nationality	
Saudis, *n* (%)	67 (98.53)
Non-Saudis, *n* (%)	1 (1.47)
Education	
Illiterate	22 (32.35)
Primary school	13 (19.12)
High school	13 (19.12)
Secondary school	7 (10.29)
Diploma degree	3 (4.41)
Bachelor’s degree	10 (14.71)
Income	
Low	13 (19.12)
Medium	44 (64.71)
High	11 (16.18)
Type of diabetes Type 1 Type 2	1 (1.47) 67 (98.53)
Number of medications	
One medication	11 (16.18)
Two medications	17 (25.00)
Three medications	35 (51.47)
Four medications	5 (7.35)
Type of medications	
Oral antidiabetics	35 (51.5)
Insulin	6 (8.8)
Combination of oral antidiabetics and insulin	27 (39.7)
Number of diabetes complications	
No complications	16 (23.53)
One complication	29 (42.65)
Two complications	18 (26.47)
Three complications	5 (7.35)

## Data Availability

The data will be available from author upon reasonable request.
